# Obituary: Prof. Dr. Albrecht M. Kellerer (1935–2022)

**DOI:** 10.1007/s00411-022-01001-4

**Published:** 2022-10-18

**Authors:** J. Chen, A. A. Friedl, E. Nekolla, W. Rühm, L. Walsh, A. Wojcik, H. Zitzelsberger

**Affiliations:** 1 Radiation Protection Bureau, Health Canada, Ottawa, ON Canada; 2 Department of Radiation Oncology, KUM University Hospital, LMU University of Munich, Munich, Germany; 3 Federal Office for Radiation Protection (BfS), Munich, Germany; 4 Helmholtz Zentrum München, Deutsches Forschungszentrum für Gesundheit und Umwelt (GmbH), Neuherberg, Oberschleißheim, Germany; 5 Department of Physics, Science Faculty, University of Zürich, Winterthurerstrasse 190, 8057 Zurich, Switzerland; 6 Centre for Radiation Protection Research, MBW Department, Stockholm University, Svante Arrhenius väg 20C, 106 91 Stockholm, Sweden


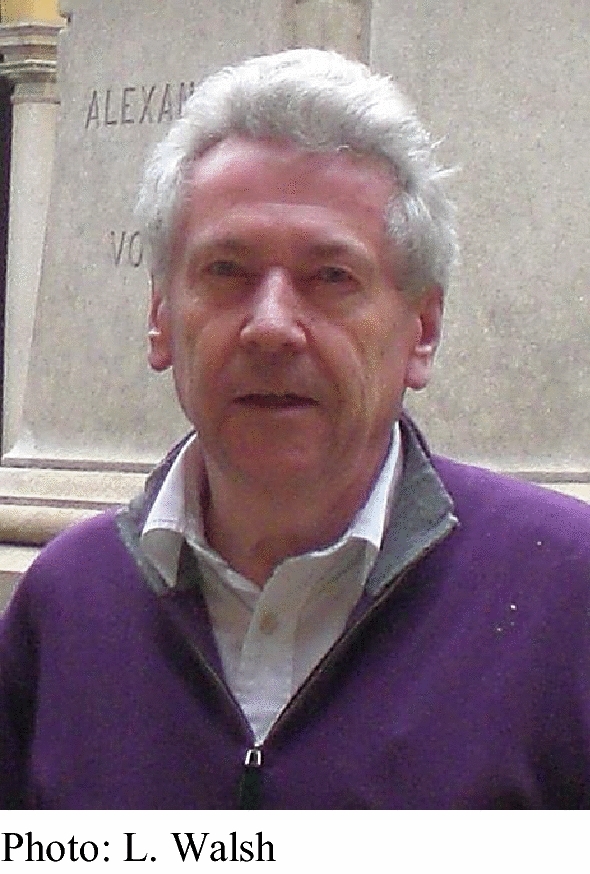


It is with deep sadness that we have to inform the readers of Radiation and Environmental Biophysics that Prof. Albrecht M. Kellerer passed away on July 31, 2022, at the age of 86. Albrecht Kellerer served as Editor-in-Chief of this journal from 1996 to 2005. In this function, he enthusiastically continued the tradition of the previous Editors-in-Chief, Boris Rajewsky, Hermann Muth, and Ulrich Hagen, by supporting radiation researchers publishing their results and sharing their knowledge with the international scientific community. Of utmost importance to him was to maintain a high scientific quality of the articles and thereby maintaining the journal, at the highest professional level.

The authors of this obituary include the current Editors-in-Chief of Radiation and Environmental Biophysics (AF, AW, WR). We are very grateful that we got the chance to continue this journal in the spirit of Albrecht Kellerer, in further developing Radiation and Environmental Biophysics as one of the leading international journals in radiation research during times of major changes including digitalization and the development of social media, and in supporting the scientific community, especially scientists new in the field, in publishing their papers. Furthermore, most of the authors had the privilege to work with Albrecht Kellerer in Munich at the Radiation Biology Institute (SBI) of the Ludwig Maximilians University (LMU). All of us are, in one way or the other, still active in the field of radiation sciences, and continue to contribute to the development of radiation science in his spirit. Clearly, without the unconditional support and his profound advice, it would have been impossible for us to develop our own careers in radiation sciences and take responsibilities in various functions on national and international levels.

Albrecht Kellerer studied physics at the LMU Munich. After his PhD, he continued at the institute of radiation biology at the LMU Munich lead by Prof. Otto Hug, where he worked for almost seven years on concepts in micro-dosimetry and dual radiation action. This early work is documented—interestingly by itself—in the first volume of Radiation and Environmental Biophysics when all articles were still published in German language (Hug and Kellerer 1963; Kellerer and Hug 1963). The following stay at Columbia University in the US turned out to be very fruitful and laid the foundation of a lifelong cooperation and friendship with Prof. Harold Rossi, one of the giants of radiation research.

Finally, early in the 1990s, Albrecht Kellerer returned to Munich to become the Director of the Radiation Biology Institute at the LMU where he worked until his retirement in 2004. Becoming the successor of his mentor Otto Hug was as if he closed the circle of his scientific career. At that time, he continued his research on radiation physics and micro-dosimetry, developed mathematical methods to be used in radiation biology, and contributed toward the assessment of radiation-related risks. The reference list below includes a number of Albrecht Kellerer’s articles published between 1996 and 2005 in Radiation and Environmental Biophysics. While this list can only partly reflect the many scientific interests Albrecht Kellerer had, it may provide at least a glimpse of the many research topics to which he contributed and where we had the honor to co-author with him.

## Jing Chen (JC)

In 1975, Albrecht Kellerer accepted an offer of the University of Würzburg, Germany, and became the Director of the Institute for Medical Radiation Science. Needless to say, that this university has a long-standing tradition in radiation sciences as it hosted the place where Wilhelm Conrad Röntgen made his ground-breaking discovery of X-rays in 1905. Albrecht Kellerer is one of the key founders of micro-dosimetry describing remarkable feature of ionizing radiation—discontinuous interaction with matter. In a book chapter “Fundamentals of Microdosimetry” published in 1985, he elegantly illustrated two important aspects of micro-dosimetry, the spatial patterns of energy deposition in the tracks of charged particles and the resulting biological effectiveness of radiation. Albrecht Kellerer led the development of the ICRP Publication 92—Relative Biological Effectiveness (RBE), Quality factor (Q), and Radiation Weighting Factor (*w*_R_) where the measurable micro-dosimetric parameter of lineal energy was introduced for more convenient computation of radiation quality factor (ICRP 2003). While actively developing the concepts of micro-dosimetry with more than hundred publications in theoretical and computational micro-dosimetry, Albrecht Kellerer also dedicated his time to the development of measurement techniques in experimental micro-dosimetry. A good example of his contribution to experimental micro-dosimetry is the development of the variance–covariance method to measure dose-mean lineal energy (the micro-dosimetric parameter closely related to radiation quality) in varying radiation fields (Kellerer and Chmelevsky 1975a, 1975b, 1975c; Kellerer and Rossi 1978; Chen et al. 1992, 1995, 1996; Chen and Kellerer 1997; Kellerer and Chen 2003; Chen and Kellerer 2006).

## Werner Rühm (WR)

In the 1990s, the dosimetry of the Japanese atomic bomb survivors, and in particular their neutron doses, was heavily debated among the international radiation research community. In fact, numerous measurements of residual radioactivity in environmental samples from Hiroshima induced by neutrons from the bomb seemed to indicate that neutron fluences and thus neutron doses were highly underestimated (by a factor of 10 or even more), in particular at those distances from the explosion where inhabitants survived the explosion. Albrecht Kellerer was very much concerned about this situation, because international regulations on radiological protection were (and by the way still are) significantly based on estimates of radiation-induced risks obtained from the atomic bomb survivors. Consequently, wrong doses to the survivors would have challenged the system of radiological protection that was in place at that time. Consequently, it was in 1997 when Albrecht Kellerer offered me (WR) to join the SBI and work on the retrospective dosimetry of the atomic bomb survivors.

To cut a long story short, studies of ^63^Ni in copper samples and ^39^Ar in granite samples both produced by fast neutrons from the Hiroshima bomb, and ^36^Cl in granite samples and ^41^Ca in tooth enamel from atomic bomb survivors both produced by moderated thermal neutrons from the bomb, were all performed in Munich as part of a large international effort on dose reconstruction. This dose reconstruction led to the Dosimetry System 2002 (Young and Kerr 2005) and finally demonstrated that the neutron doses to the survivors in Hiroshima were largely correct and the deduced radiation risk estimates reliable. Thanks to the continuous interest and tireless support of Albrecht Kellerer that this long-lasting project could be realized (Rühm et al. 1998, 2000, 2007, 2010; Kellerer and Rühm 2002; Huber et al. 2003, 2005; Straume et al. 2003, 2004; Nolte et al. 2005, 2006; Hoshi et al. 2008; Wallner et al. 2010).

## Elke Nekolla (EN) and Linda Walsh (LW)

It was in the early 1990s when I (EN), a young mathematician, joined the SBI to help evaluate a radiation epidemiology study on the effects of the short-lived alpha-particle emitter ^224^Radium. I came directly from the university, where I had mainly dealt with pure mathematics, without any real plan regarding my career path. At the SBI, I immediately realized that interdisciplinary work in the field of radiation protection would be the right thing for me to do. In particular I was interested in radiation epidemiology, which, at that time, had not yet been given much attention in Germany. The study I was recruited to evaluate was the so-called “Spiess Study”, which followed the health of individuals who had received numerous injections of ^224^Radium mainly between 1945 and 1955 for the treatment of tuberculosis, ankylosing spondylitis and some other diseases. Tragically, not only was this a completely ineffective therapy, but malignant bone tumors appeared just a few years after treatment in a temporal wave that peaked around eight years after exposure (57 cases vs. less than one case expected). Albrecht Kellerer and colleagues had already intensively researched these late effects and found, among other things, an unexpected “inverse dose-rate effect” (or “reverse protraction factor”, i.e., the longer the period of therapy—at equal cumulative dose—the higher the risk of a radiation-induced bone tumor) (Chmelevsky et al. 1990). After a longer time of follow-up, other types of cancer appeared—this was the time when I had the opportunity to participate in the study (Nekolla et al. 2010).

In the years up to 2000, Albrecht Kellerer had been thinking very deeply about the γ- and neutron dosimetry for the Hiroshima and Nagasaki A-bomb survivors cohort and the influence that certain dosimetric characteristics may have on the cancer risks per unit organ equivalent dose. He was convinced that the late radiation effects were almost fully being attributed to the γ-doses due to too low a weighting of neutrons in the equivalent dose and a too high body shielding of the neutrons from referencing the cancer risks with respect to the equivalent dose to the deeply lying colon.

Subsequently to the earlier work with EN, he offered a post-doc position (LW) in 2000 to further the work on these aspects. Between 2000 and 2005, the LMU sub-group (LW, WR, EN) published several papers with Albrecht Kellerer that refined the usual Radiation Effects Research Foundation approach, by developing new methods to either consider all solid cancer risks associated with γ-doses and neutron doses separately (Kellerer et al. 2001, Kellerer and Walsh 2001, Kellerer and Walsh 2002, Kellerer et al. 2002), or apply organ-specific doses (Walsh et al. 2004a, 2004b) and some indications were found for a higher neutron relative biological effectiveness (RBE) with respect to gammas, than previously assumed (Kellerer et al. 2006). Such indications are important because, for example, a large fraction of proton therapy patients receive an additional neutron dose as an unwanted by-product. In January 2012, an international research project started under the seventh framework program of the European Union, FP-7-EU-ANDANTE (Multidisciplinary evaluation of the cancer risk from neutrons relative to photons using stem cells and the analysis of second malignant neoplasms following pediatric radiation therapy), and Albrecht Kellerer came out of retirement to act as an advisor to this project (the photo above was taken in front of the statue of Alessandro Volta during the ANDANTE kick-off meeting at the University of Pavia).

## Anna A. Friedl (AAF)

In spite of his physicist’s upbringing, Albrecht Kellerer enthusiastically supported the beginning of molecular radiation biology. For a quantitative analysis of repair of DNA double-strand breaks (DSB) in the 1990s, pulsed-field gel electrophoresis became the tool of choice and Albrecht Kellerer helped me (AAF) and another PhD student, Alfred Kraxenberger, in developing computer-simulation evaluation methods that allowed us not only to quantitate induction and repair of DSB, but also to analyze their spatial distribution after low and high LET irradiation (Kraxenberger et al. 1994, 1998, Friedl and Kellerer 2001). Albrecht Kellerer never co-authored papers to which he did not contribute scientifically. Therefore, many people are not aware of his huge support for the early studies of DSB repair mechanisms performed in his institutes at LMU and GSF-Forschungzentrum.

## Horst Zitzelsberger (HZ)

Albrecht Kellerer was also interested in exploring the health consequences of the Chernobyl reactor accident in 1986 in terms of radiation epidemiology and aforementioned molecular radiation biology (Ivanov et al. 1996, 1998; Gapanovich et al. 2001; Kellerer 2002a, b). In addition to childhood leukemia, also childhood thyroid cancer was among his main scientific interests. He generously supported my own studies and academic career (HZ) on the relationship between radiation exposure and molecular genetic and cytogenetic changes in childhood thyroid cancer with the aim to unravel molecular mechanisms and to identify radiation-specific molecular markers for an improvement of thyroid cancer risk assessment (Lehmann et al. 1996; Zitzelsberger et al. 1999; Smida et al. 1999; Richter et al. 1999; Salassidis et al. 2000; Lohrer et al. 2001). One of Albrecht Kellerer’s major achievements in this field was to build a bridge between epidemiological findings and molecular biological understanding of radiation-induced carcinogenesis. His overarching and broad knowledge in many disciplines of radiation research were of inestimable value for all young investigators in this complex and interdisciplinary scientific area.

## References

[CR1] Chen J, Kellerer AM (1997). Calculation of radial dose distributions for heavy ions using a new analytical approach. Radiat Prot Dosim.

[CR2] Chen J, Kellerer AM (2006). Proximity functions of electrons from 100eV to 10MeV. Radiat Prot Dosim.

[CR3] Chen J, Roos H (1992). Kellerer AM (1992) Microdosimetry of diagnostic x-rays: applications of the variance-covariance method. Radiat Res.

[CR4] Chen J, Kellerer AM, Rossi HH (1995). On the revised concept of linear energy transfer. Radiat Environ Biophys.

[CR5] Chen J, Nekolla E, Kellerer AM (1996). A comparative study of microdosimetric properties of x-, gamma- and beta rays. Radiat Environ Biophys.

[CR6] Chmelevsky D, Spiess H, Mays CW, Kellerer AM (1990). The reverse protraction factor in the induction of bone sarcomas in radium-224 patients. Radiat Res.

[CR7] Friedl AA, Kellerer AM (2001). Comments on 'Underestimation of the small residual damage when measuring DNA double-strand breaks (DSB): is the repair of radiation-induced DSB complete?'. Int J Radiat Biol.

[CR8] Gapanovich VN, Iaroshevich RF, Shuvaeva LP, Becker SI, Nekolla EA, Kellerer AM (2001). Childhood leukemia in Belarus before and after the Chernobyl accident: continued follow-up. Radiat Environ Biophys.

[CR9] Hoshi M, Endo S, Tanaka K, Ishikawa M, Straume T, Komura K, Rühm W, Nolte E, Huber T, Nagashima Y, Seki R, Sasa K, Sueki K, Fukushima H, Egbert SD, Imanaka T (2008). Intercomparison study on ^152^Eu gamma ray and ^36^Cl AMS measurements for development of the new Hiroshima-Nagasaki atomic bomb dosimetry system 2002 (DS02). Radiat Environ Biophys.

[CR10] Huber T, Rühm W, Hoshi M, Egbert SD, Nolte E (2003). ^36^Cl measurements in Hiroshima granite samples as part of an international intercomparison study: results from the Munich group. Radiat Environ Biophys.

[CR11] Huber T, Rühm W, Kato K, Egbert SD, Kubo F, Lazarev V, Nolte E (2005). The Hiroshima thermal-neutron discrepancy for ^36^Cl at large distances. Part I: new ^36^Cl measurements in granite samples exposed to A-bomb neutrons. Radiat Environ Biophys.

[CR12] Hug O, Kellerer AM (1963). Zur Interpretation der Dosiswirkungsbeziehungen in der Strahlenbiologie. Radiat Environ Biophys.

[CR13] ICRP (2003) Relative biological effectiveness (RBE), Quality Factor (*Q*), and radiation weighting factor (*w*_R_). ICRP publication 92. Ann ICRP 33(4):1–117 10.1016/s0146-6453(03)00024-1 14614921

[CR14] Ivanov EP, Tolochko GV, Shuvaeva LP, Becker S, Nekolla E, Kellerer AM (1996). Childhood leukemia before and after the Chernobyl accident. Radiat Environ Biophys.

[CR15] Ivanov EP, Tolochko GV, Shuvaeva LP, Ivanov VE, Iaroshevich RF, Becker S, Nekolla E, Kellerer AM (1998). Infant leukemia after the Chernobyl accident. Radiat Environ Biophys.

[CR16] Kellerer AM (1975). Chmelevsky D (1975c) Concepts of microdosimetry – III. Mean values of the microdosimetric distributions. Radiat Environ Biophys.

[CR17] Kellerer AM (2002). Beyond Chernobyl: the new Russian studies in perspective. Radiat Environ Biophys.

[CR18] Kellerer AM, Chen J (2003). Comparative microdosimetry of photo- and Compton electrons – an analysis in terms of generalized proximity functions. Radiat Res.

[CR19] Kellerer AM, Chmelevsky D (1975). Concepts of microdosimetry – I. Quantities. Radiat Environ Biophys.

[CR20] Kellerer AM, Chmelevsky D (1975). Concepts of microdosimetry – II. Probability distributions of the microdosimetry variables. Radiat Environ Biophys.

[CR21] Kellerer AM, Hug O (1963). Zur Kinetik der Strahlenwirkung. Radiation Environ Biophys.

[CR22] Kellerer AM, Rossi HH (1978). A generalized formulation of dual radiation action. Radiat Res.

[CR23] Kellerer AM, Rühm W (2002). Evolution in zigzag – the changing state of A-bomb dosimetry. J Radiol Protect.

[CR24] Kellerer AM, Walsh L (2001). Risk estimation for fast neutrons with regard to solid cancer. Radiat Res.

[CR25] Kellerer AM, Walsh L (2002). Solid cancer risk coefficient for fast neutrons, in terms of effective dose. Radiat Res.

[CR26] Kellerer AM, Nekolla E, Walsh L (2001). On the conversion of solid cancer excess relative risk into lifetime attributable risk. Radiat Environ Biophys.

[CR27] Kellerer AM, Walsh L, Nekolla EA (2002). Risk coefficient for γ-rays with regard to solid cancer. Radiat Environ Biophys.

[CR28] Kellerer AM, Rühm W, Walsh L (2006). Indications of the neutron effect contribution in the solid cancer data of the A-bomb survivors. Health Phys.

[CR29] Kraxenberger A, Friedl AA, Kellerer AM (1994). Computer simulation of pulsed field gel runs allows the quantitation of radiation-induced double-strand breaks in yeast. Electrophoresis.

[CR30] Kraxenberger F, Weber KJ, Friedl AA, Eckardt-Schupp F, Flentje M, Quicken P, Kellerer AM (1998). DNA double-strand breaks in mammalian cells exposed to gamma-rays and very heavy ions. Fragment-size distributions determined by pulsed-field gel electrophoresis. Radiat Environ Biophys.

[CR31] Lehmann L, Zitzelsberger H, Kellerer AM, Braselmann H, Kulka U, Georgiadou-Schumacher V, Negele T, Spelsberg F, Demidchik E, Lengfelder E, Bauchinger M (1996). Chromosome translocations in thyroid tissues from Belarussian children exposed to radioiodine of the Chernobyl accident, measured by FISH-painting. Int J Radiat Biol.

[CR32] Lohrer HD, Braselmann H, Richter HE, Jackl G, Herbeck J, Hieber L, Kellerer AM, Bauchinger M (2001). Instability of microsatellites in radiation-associated thyroid tumours with short latency periods. Int J Radiat Biol.

[CR33] Nekolla EA, Walsh L, Spiess H (2010). Incidence of malignant diseases in humans injected with radium-224. Radiat Res.

[CR34] Nolte E, Huber T, Rühm W, Kato K, Lazarev V, Schultz L (2005). The Hiroshima thermal-neutron discrepancy for ^36^Cl at large distances. Part II: natural in situ production as a source. Radiat Environ Biophys.

[CR35] Nolte E, Rühm W, Loosli HH, Tostikhin I, Kato K, Huber TC, Egbert SD (2006). Measurements of fast neutrons in Hiroshima by use of ^39^Ar. Radiat Environ Biophys.

[CR36] Young RW, Kerr GD, Report of the Joint US-Japan Working Group (2005). Reassessment of the atomic bomb radiation dosimetry for Hiroshima and Nagasaki: Dosimetry System.

[CR37] Richter HE, Lohrer HD, Hieber L, Kellerer AM, Lengfelder E, Bauchinger M (1999). Microsatellite instability and loss of heterozygosity in radiation-associated thyroid carcinomas of Belarussian children and adults. Carcinogenesis.

[CR38] Rühm W, Kellerer AM, Korschinek G, Faestermann T, Knie K, Rugel G, Kato K, Nolte E (1998). The dosimetry system DS86 and the neutron discrepancy in Hiroshima - historical review, present status, and future options. Radiat Environ Biophys.

[CR39] Rühm W, Knie K, Rugel G, Marchetti AA, Faestermann T, Wallner C, McAninch JE, Straume T, KorschinekG,  (2000). Accelerator mass spectrometry of ^63^Ni at the Munich tandem laboratory for estimating fast neutron fluences from the Hiroshima atomic bomb. Health Phys.

[CR40] Rühm W, Carroll KL, Egbert SD, Faestermann T, Knie K, Korschinek G, Martinelli RE, Marchetti AA, McAninch JE, Rugel G, Straume T, Wallner A, Wallner C, Fujita S, Hasai H, Hoshi M, ShizumaK,  (2007). Neutron-induced ^63^Ni in copper samples from Hiroshima and Nagasaki: a comprehensive presentation of results obtained at the Munich maier-leibnitz laboratory. Radiat Environ Biophys.

[CR41] Rühm W, Wallner A, Cullings H, Egbert SD, El-Faramawy N, Faestermann T, Kaul D, Knie K, Korschinek G, Nakamura N, Roberts J, Rugel G (2010). ^41^Ca in tooth enamel. Part II: a means for retrospective biological neutron dosimetry in atomic-bomb survivors. Radiat Res.

[CR42] Salassidis K, Bruch J, Zitzelsberger H, Lengfelder E, Kellerer AM, Bauchinger M (2000). Translocation t(10;14)(q11.2;q22.1) fusing the kinetin to the RET gene creates a novel rearranged form (PTC8) of the RET proto-oncogene in radiation-induced childhood papillary thyroid carcinoma. Cancer Res.

[CR43] Smida J, Salassidis K, Hieber L, Zitzelsberger H, Kellerer AM, Demidchik EP, Negele T, Spelsberg F, Lengfelder E, Werner M, Bauchinger M (1999). Distinct frequency of ret rearrangements in papillary thyroid carcinomas of children and adults from Belarus. Int J Cancer.

[CR44] Straume T, Rugel G, Marchetti AA, Rühm W, Korschinek G, McAninch JE, Caroll KL, Egbert SD, Faestermann T, Knie K, Martinelli RE, Wallner A, Wallner C (2003). Measuring fast neutrons in Hiroshima at distances relevant to atomic-bomb survivors. Nature.

[CR45] Straume T, Rugel G, Marchetti AA, Rühm W, Korschinek G, McAninch JE, Carroll K, Egbert SD, Faestermann T, Knie K, Martinelli RE, Wallner A, Wallner C, Fujita S, Shizuma K, Hoshi M, Hasai H (2004). Addendum: measuring fast neutrons in Hiroshima at distances relevant to atomic-bomb survivors. Nature.

[CR46] Wallner A, Rühm W, Rugel G, Nakamura N, Arazi A, Faestermann T, Knie K, Maier HJ, Korschinek G (2010). ^41^Ca in tooth enamel. Part I: a biological signature of neutron exposure in atomic-bomb survivors. Radiat Res.

[CR47] Walsh L, Rühm W, Kellerer AM (2004). Cancer risk estimates for γ-rays with regard to organ specific doses Part I: all solid cancers combined. Radiat Environ Biophys.

[CR48] Walsh L, Rühm W, Kellerer AM (2004). Cancer risk estimates for γ-rays with regard to organ specific doses Part II: site specific solid cancers. Radiat Environ Biophys.

[CR49] Zitzelsberger H, Lehmann L, Hieber L, Weier HU, Janish C, Fung J, Negele T, Spelsberg F, Lengfelder E, Demidchik EP, Salassidis K, Kellerer AM, Werner M, Bauchinger M (1999). Cytogenetic changes in radiation-induced tumors of the thyroid. Cancer Res.

